# Resource constraints, patient emotions, and therapy: experiences of healthcare providers in the screening and management of gestational diabetes mellitus in northern Ghana

**DOI:** 10.1186/s12913-025-12262-2

**Published:** 2025-02-05

**Authors:** Mohammed Bukari, Abdulai Abubakari, Hawa Malechi, Thomas Hinneh, Humphrey Garti, Faith Agbozo

**Affiliations:** 1 Department of Nutritional Sciences, School of Allied Health Sciences, University for Development Studies, P.O. Box TL 1883, Tamale, Ghana; 2 Department of Social and Behavioral Change, School of Public Health, University for Development Studies, P.O. Box TL1350, Tamale, Ghana; 3 Department of Global and International Health, School of Public Health, University for Development Studies, P.O. Box TL1350, Tamale, Ghana; 4 Department of Obstetrics and Gynaecology, Tamale Teaching Hospital, P.O. Box TL 16, Tamale, Ghana; 5 Johns Hopkins University School of Nursing, Baltimore, USA; 6 Heidelberg Institute of Global Health, Heidelberg University, Heidelberg, 69120 Germany; 7 Department of Family and Community Health, University of Allied Health Sciences, Private Mail Box 31, Ho, Ghana

**Keywords:** Gestational diabetes, Screening, Management, Experiences, Healthcare providers, Ghana

## Abstract

**Background:**

Studies on healthcare providers’ experiences in screening and managing Gestational Diabetes Mellitus (GDM) are rare in northern Ghana. Understanding the experiences of healthcare providers in the screening and management of GDM has the potential to identify best practices to improve GDM care. Hence, this study sought to explore the experiences of healthcare providers in screening and managing GDM.

**Materials and methods:**

This was a qualitative study of five key informants involved in the screening and management of GDM, specifically an obstetrician, a dietician/nutritionist, a nurse, and two midwives in the northern region of Ghana. Face-to-face interviews were conducted with the aid of an interview guide, and the data were analysed via thematic analysis. We ensured trustworthiness through reflexivity, member checking, peer-debriefing, providing rich text, and keeping an audit trail.

**Results:**

Two main themes and six subthemes were generated. The first theme was resource-centred experiences, which explored events at the Antenatal Care (ANC) and the challenges in GDM screening and management, with subthemes namely: ANC setups, inadequate resources, and non-compliance. The second theme was care-centred experiences, which explored patient emotions during GDM care, and the care they receive post-GDM diagnosis, with subthemes namely: raw emotions, non-pharmacological therapy, and pharmacotherapy.

**Conclusion:**

Findings suggest the need for health system strengthening through the training and recruitment of dedicated skilled personnel, as well as government intervention through policy revision to cover the cost of GDM diagnostic tests. Healthcare providers should also consider the psycho-emotional impact of diagnosis when managing GDM. The findings could be relevant for health policy, planning, advocacy, and social and behaviour change communication aimed at effective screening and management of GDM.

## Introduction

Gestational Diabetes Mellitus (GDM) refers to varying levels of glucose intolerance that are first detected during pregnancy [1]. The prevalence of GDM has risen over the years, with approximately 90% of cases occurring in Low- and Middle-Income Countries (LMICs), including Ghana [2]. The prevalence of GDM in Ghana is within the range of 10–14% [3, 4]; some of the risk factors for GDM include; maternal age, gravidity, parity, family history of GDM, blood pressure, [3] and obesity [4].

Three approaches are used in screening for GDM, namely, universal screening, selective screening, and risk factor stratification [5]. According to Agbozo et al. [6], universal screening involves screening all pregnancies regardless of their glycaemic status and subsequently booking them for diagnostic tests for GDM, whereas selective screening involves assessments conducted at the first prenatal visit. Risk factor screening involves risk factor stratification based on the severity of risk [6]. Universal screening is preferred despite concerns about the financial burden it places on the health system [6]. On the diagnostic test, the 75-g oral glucose tolerance test (OGTT) at 24–28 weeks is regarded as the gold standard [7]. Nonetheless, the WHO criteria focuses on prognostic accuracy, and not diagnostic accuracy, and does not consider any test the gold standard [8].

Several challenges affect the screening and management of GDM. Some studies in Africa have identified patient-level factors such as unemployment, limited access to healthy foods, as well as health facility-level factors such as poor health insurance coverage and unreliable transportation network as key barriers for management of GDM in Africa [5, 9]. Sahu et al. [10] also documented factors such as delays in seeking healthcare, unmet training needs for healthcare providers, long waiting hours, limited diagnostic capacity, and lack of follow-up, as barriers to screening and managing GDM. These challenges cumulatively underscore the need to implement multi-sectoral approaches to improve the continuum of GDM care in low resource settings like Ghana.

Meanwhile, better maternal and foetal health outcomes are a result of managing GDM during pregnancy [11, 12]. Studies have shown that monitoring blood sugar levels and making dietary and lifestyle modifications constitute the initial management of GDM; pharmacologic therapy, including the use of metformin, glyburide, or insulin, is considered only when the initial management methods fail [13, 14]. The American Diabetes Association’s standards of medical care for patients with diabetes also recommend that when medical nutrition therapy, exercise, and glucose monitoring are ineffective at maintaining a normal blood glucose level, insulin may be given as a last resort [15]. A study in Ghana concluded that educating women diagnosed with GDM about the condition, and enlisting the help of close relatives were essential components of the nursing management of women with GDM [16].

Despite being discredited by the National Institute of Health Care Excellence, glycosuria analysis is still used in screening for GDM. For instance, in Ghana, dipstick glycosuria analysis is conducted at each Antenatal Care (ANC) visit, followed with fasting blood glucose tests at 28–32 weeks of gestation, and at birth [17]. It is worth noting that the use of glycosuria as a routine screening test would reduce the chances of early detection and treatment of GDM because of false-negative cases; screening and diagnostic protocols are unrestricted and mostly dependent on the level of care, and hence, diagnostic decisions are normally made based on maternal risk factors [3].

Ineffective screening and management of GDM leads to delivery complications and adverse maternal and foetal outcomes [18]. Exploring the experiences of Healthcare Providers (HCPs) in the screening and management of GDM has the potential to identify best practices for GDM care. However, the few studies available in Ghana have focused on the prevalence [4], and diagnostic accuracy of GDM screening tools [3] at the expense of healthcare providers’ experiences. This study sought to explore the experiences of HCPs in the screening and management of GDM.

## Methods

### Study area

The study was conducted in four health facilities in the Tamale metropolis. The Ghanaian health system is a three-tiered system, namely: primary, secondary and tertiary care. Three of the facilities sampled in this study were at the secondary level (Tamale Central Hospital (TCH), Tamale West Hospital (TWH) and SDA (Seventh-Day Adventists) Hospital), and one was at the tertiary level (Tamale Teaching Hospital (TTH)). According to the 2010 population census, the total fertility rate of the Tamale metropolis is 2.8, the crude birth rate is 21.2/1000, and the general fertility rate is 79.9/1000 among women aged 15–49 years [19].

### Study design, study population, sampling, and sampling techniques

The study was a phenomenological study. The study population included HCPs involved in the screening and management of GDM, specifically: an obstetrician, a dietician/nutritionist, a nurse, and two midwives. Purposive sampling strategy was adopted in selecting both the health facilities and the healthcare providers. The idea of purposive sampling was to achieve saturation, enhance richness and diversity of the study population [20]. Primary emphasis was on experience, expertise, and variety. The sample size was selected on the basis of data saturation, and was determined after five key informant interviews. Saturation was achieved when data collection no longer yielded new insights or issues, and no new themes emerged from the interviews [21]. The facilities selected had ANC setups that included nutritionists, nurses, midwives, medical officers, and a laboratory attached to the ANC. Facility heads or officers in charge of the various facilities were initially interviewed to identify key informants or asked to recommend experienced personnel involved in the screening and management of GDM within their respective facilities. Only HCPs working in the selected facilities and involved in the screening and management of GDM were included in the study.

### Data collection

The data were obtained through face-to-face key-informant interviews. Interview guides were designed based on the literature and internal discussions to elicit information on experiences in screening and managing GDM. For example, earlier evidence from Africa has identified barriers to screening and diagnosis, hindrances to implementing management interventions, and experiences of women regarding GDM diagnosis and management [5]. Additionally, a study in India also identified some perceptions of HCPs regarding GDM care [10]. Hence, the interview guide explored perceptions of HCPs on how pregnant women felt about being diagnosed with GDM, the screening process, GDM care*,* and the challenges they face in GDM care. Examples of questions contained in the interview guide were as follows: *What challenges do you face in screening for GDM? What are the feelings/emotions of women and their families after learning about their GDM status? What treatment does women receive after GDM diagnosis? What challenges do you think diagnosed women face, and how does it affect you? Do you experience noncompliance? If yes, what are the reasons for noncompliance? What are the most important resources required to effectively screen for and manage GDM?*

The interviews were conducted by two community nutritionists with Bachelor’s degree, proficient in English language with prior experience in qualitative data collection: one administered the questionnaire while the other took the notes. The interviewers were trained on the data collection instrument. The interviews were recorded using audio-recorders, and lasted from 45 minutes to 1 hour 20 minutes.

### Trustworthiness

To enhance the trustworthiness of this study, we focused on addressing issues regarding credibility, transferability, dependability, and confirmability [22, 23]. To ensure the credibility, study participants were provided with pre-publication copies of the manuscript and asked to provide feedback on the accuracy of the results presented; all of them were satisfied with the information presented to them. On the issue of transferability, we provided information on the study area, background information about the participants, and rich text about the participants’ experiences. With regard to dependability and confirmability, we engaged in peer-debriefing and member checking throughout the research process. We also kept an audit trail while engaging in regular reflexivity throughout this study.

### Reflexivity

We recognize that our position as trained HCPs and academics has shaped our thinking about the topic of healthcare providers’ experience in the screening and management of GDM. Hence, we approached the topic from an insider-outsider position, the insider-outsider position refers to the dual role of a researcher who is both a member of a group being studied and an external observer [24]. We regularly engaged in bracketing to minimize any bias we would have introduced into the study. We acknowledge that social dynamics and power structures exist within the healthcare system. Hence, we investigated the potential impact of health policies and resource allocation on the experiences of HCPs. For example, there was an instance when one participant asked if we were going to provide the resources they lacked. We could only explain that we were active collaborators in the production of knowledge with the hope that the knowledge produced reaches the appropriate personnel or institutions. In all situations, we tried to create an open and respectful environment that encouraged study participants to freely express their views while addressing any power differences that arose during interviews. Drawing on our Ghanaian identities, we built ethical and deferential relationships with the study participants.

### Data analysis

We used Braun and Clarke’s six steps for thematic analysis [25]. Key informant interviews were organized, and repeatedly reviewed for familiarization and transcribed verbatim. We generated initial codes for the data and subsequently identified themes. These themes were reviewed and explained. MB and AA manually performed the coding, and FA reviewed the codes; all discrepancies were resolved through consensus while taking into account the dataset used to generate the codes. We used a blend of deductive and inductive coding; initial codes were generated using subtopics from the interview guide, and data from the subtopics were then used to generate subthemes and main themes.

## Results

The total number of HCPs interviewed was five; the minimum educational qualification for the study participants was a diploma in nursing, and the highest were the specialization of a medical officer in obstetrics and gynecology, and a nutritionist obtaining an advanced degree in dietetics. Their ages ranged from 28 to 44 years (see Table 1).

**Table 1:** Profile of the study participants

Age	Designation	Qualification	Experience in years
20–30	Staff Nurse	Diploma in Nursing	5–10
20–30	Midwifery officer	BSc Midwifery	3–5
31–40	Obstetrician	MBBS, OBG	5–10
31–40	Dietician/Nutritionist	BSc Community Nutrition, MPhil Dietetics	10–15
41–50	Senior Midwifery officer	BSc Midwifery	15–20

### Themes and subthemes

Two main themes and six subthemes were generated. The two main themes were resource-centred experiences and care-centred experiences. The six subthemes were: ANC setups, inadequate resources, non-compliance, raw emotions, non-pharmacological therapy, and pharmacotherapy (Table 2).

**Table 2:** Themes and subthemes

Theme	Subtheme
Resource-centred Experiences	Antenatal Care (ANC) setups
	Inadequate resources
	Non-compliance
Care-centred Experiences	Raw emotions
	Non-pharmacological therapy
	Pharmacotherapy

### Resource-centred experiences

This theme focused on events at the ANC and the various challenges HCPs face in screening and managing GDM. It comprised three subthemes, namely: ANC setups, inadequate resources, and non-compliance.

### ANC setups

The obstetrician outlined that during ANC, medical history and physical examinations were conducted, and laboratory tests were requested from laboratories. Midwives performed vital signs and conducted protein and glucose in urine tests. Screening and diagnosis were performed after 20 weeks of gestation. Women were counselled before and after screening, and those with abnormal results were counselled and referred to diabetic clinics or obstetrics and gynaecology units. GDM was best diagnosed at approximately 24–28 weeks of pregnancy after three consecutive fasting blood glucose or glycated haemoglobin tests. The explanation below was what one Healthcare Provider (HCP) had to give:

*“From the history, physical examination and laboratory test, particularly history, if we find anything unusual, then we are more embolden to perform further tests.”* (# Obstetrician)

The Obstetrician explained*, “Women usually undergo several tests; urine (rapid) tests and positive cases are referred for further tests, which include fasting blood glucose tests and/or glycated haemoglobin (HBA1c) tests.”* (#Obstetrician)

The Obstetrician further offered that *“elsewhere, all women get an OGTT, but it is not done here because of cost. So, we do a history first, and then, when we find risk factors, we ask them to do OGTT. So, the screening helps to identify those who are at risk of GDM.”* (#Obstetrician)

The Obstetrician added that, *“We pick up certain risk factors; person’s weight, previous history of GDM, current history of diabetes, stillbirth and any foetal anomalies in previous pregnancy are looked at.”* (#Obstetrician)

One of them added that on clinic days, screening was free as a result of the free maternal care policy and institutionalization of a National Health Insurance Scheme (NHIS). Patients were only made to pay for HBA1c test, and when they visited ANC on days other than their designated clinic days. Another added that the urine test was free, but random glucose cost 5 Ghana cedis (0.49 USD equivalent). Below were some of their explanations:

“*On clinic days, tests and screenings are free as a result of free maternal care policy and health insurance. Payment is for HBA1c test only and when patients come on days other than the clinic days.”* (# Dietician/Nutritionist)

The Midwife explained, *“The urine test is usually free, but there is a random glucose cost, Ghc 5, which is used to buy test strips.”* (# Midwife)

And added that, *“Screening for GDM is done every day, booking is done on monthly basis in the second trimester of pregnancy." (# Midwife)*

### Inadequate resources

All HCPs conceded that they faced challenges in screening for GDM. The challenges were: long waiting queues at laboratories, high cost of OGTT, heavy workload, inadequate equipment, and personnel to screen for GDM. Below were some of their responses:

*“Women sometimes come in at a time when laboratories have closed. Cost is another challenge; the OGTT is not covered by health insurance, and women have to pay for it, sometimes you request for it this week, and the woman would bring it 2–3 weeks later.”* (# Obstetrician)

*“Long waiting queues at laboratories, when women are stressed, they abscond.”* (# Dietician/Nutritionist)

*“The main challenge is usually inadequate test strips.”* (# Midwife)

The Dietician/Nutritionist added that*, “Cases are monitored and counselled, but due to heavy workload, follow-up is usually a challenge.*” (# Dietician/Nutritionist)

HCPs mentioned test strips and dedicated staff as important resources required for effective screening and management of GDM. Some of their responses were as follows:

*“Tools needed for screening include weight and height measuring devices, protein and glucose in urine dipsticks, glucose test strips and dedicated staff who would take their time to take good medical history and do a thorough physical examination.”* (# Obstetrician)

*“GDM should be diagnosed at 24–28 weeks. The reasons for delayed diagnosis include a lack of technical personnel and equipment for performing the test.”* (# Dietician/Nutritionist)

The Dietician/Nutritionist said: *“The most important things are that healthcare providers are available, and there should be an increase in the number of dieticians at health facilities*” (# Dietician/Nutritionist).

### Non-compliance

HCPs admitted that they experienced issues of non-compliance. Patients did not adhere to treatment regimen due to poverty, scarcity of foods prescribed by dieticians (as certain foods became costly or/and inaccessible in the dry season), and inadequate financial and emotional support from family members. Some of their responses have been captured below:

*“Women having to buy glucometers and test strips for regular monitoring come at a discomfort because of cost. So, sometimes we have to admit them for better control of blood sugar, and it comes at a discomfort to them; it becomes challenging, and they start threatening to go home.”* (# Obstetrician)

The obstetrician added, *“If you want to put them on insulin, those without fridges struggle.” (# Obstetrician)*

The Dietician/Nutritionist explained, “*Yes, we experience issues of noncompliance. The reasons for noncompliance include poverty, seasonal availability of fruits and vegetables, hormonal changes and lack of support from family members*.” (# Dietician/Nutritionist)

*“Yes, they are issues of noncompliance. Sometimes they throw medications away and eat all that you ask them not to eat and come to you with issues, and the reason is that women sometimes become fed-up with treatment; sometimes what they are supposed to eat, they don’t like it.”* (# Obstetrician)

### Care-centred experiences

This theme focuses on the kind of emotions patients expressed when diagnosed with GDM, and the care they received post-GDM diagnosis. It comprised three subthemes, namely: raw emotions, non-pharmacological therapy, and pharmacotherapy.

### Raw emotions

With respect to the feelings/emotions expressed by women and their families after learning about their GDM status, the HCPs said that women were usually worried, shocked, confused or uncertain about how they felt while others were unbothered. They added that those without family support suffered emotionally. These were some of the responses provided by HCPs:

*“Well, it depends on the person’s appreciation of what the condition is, because for some, they truly don’t understand, so they are not perturbed but those who have an understanding that this disease can affect every other part of their bodies—they tend to be quite worried, worried about the growing baby and themselves and then their future even after having the baby.”* (# Obstetrician)

One of the HCP highlighted that some of the women were *“Shocked when information is not well presented to women”* (# Nurse)

While another indicated, *“Some of the main challenges faced include financial challenges, emotional trauma and anxiety. The drugs are not easy to take.”* (# Midwife)

On financial and emotional support, one of them said, “*Hormonal changes and lack of support from family members leads to emotional trauma*.” (# Dietician/Nutritionist)

Another added that, *“Women with GDM do not receive support* [emotional and/or financial from family members]*”* (# Nurse)

### Non-pharmacological therapy

The treatment for GDM began with counselling. Women were counselled on the disease, their dietary intake, and encouraged to exercise. Below are excerpts:

*“GDM is caused by unhealthy diets, physiological changes, especially hormonal changes, genetic factors/family history, physical inactivity during pregnancy and low patronage of preconception care. To prevent GDM, patients should increase patronage of preconception care, eat healthy diets and regularly screen for chronic conditions. There should be early nutritional care.”* (# Dietician/Nutritionist)

*“We usually take them* [patients] *through counselling for them to understand the disease they have.”* (# Obstetrician)

“*Patients who can afford glucometers are asked to buy and monitor their blood glucose regularly and report to dieticians or nutrition providers (the test should be performed at least two times daily).”* (# Dietician/Nutritionist)

*“Some of the dietary approaches we use include the DASH* [Dietary Approaches for Stopping Hypertension] *diet, my plate method, and the 4-star diet. We also encourage them to avoid refined foods, engage in self-care behaviours and patronize preconception care”* (# Dietician/Nutritionist)

The Dietician/Nutritionist added that the pregnant women diagnosed with GDM were encouraged to “*Shift from Western diets to local foods, fruits and vegetables, physical activity and avoid refined foods*” (# Dietician/Nutritionist)

*“We also do dietary counselling to modulate food for them and help them to identify foods that have a high glycaemic index, which they are always advised not to take. We also encourage them to exercise.”* (# Obstetrician)

### Pharmacotherapy

Oral medications, specifically metformin and insulin, were the last line treatment approach when managing or treating GDM. Below is an excerpt from one of the participants:

“*We use oral medications; in pregnancy, metformin is good, and when we are not able to control enough, we add* insulin.” (# Obstetrician)

Another added, *“Women with GDM are usually giving metformin”* (#Midwife)

## Discussion

This study sought to explore the experiences of HCPs in the screening and management of GDM. Two main themes and six subthemes were generated. The first theme was resource-centred experiences, which explored events at the ANC, and the challenges in GDM screening and management, with three subthemes namely: ANC setups, inadequate resources, and noncompliance. The second theme was care-centred experiences, which delved into patients’ emotions during GDM diagnosis, and the care they received post-GDM diagnosis, with three subthemes namely: raw emotions, non-pharmacological therapy, and pharmacotherapy.

On resource-centred experiences, this study noted that ANC is an important setup in screening for GDM; all pregnant women are screened, and positive cases are referred to laboratories for further testing. The free maternal health policy and national health insurance policies have been essential for accessing health care; however, these policies do not cover tests for GDM diagnosis, specifically the OGTT, which is recommended as a confirmatory test by many of the criteria used in the diagnosis of GDM [7, 8, 26]. This indicates the need for GDM diagnostic tests, particularly the OGTT, to be covered by the NHIS. Since, a delay in diagnosis could worsen the condition, and thus put both the mother and the baby at risk of complications. Some of the reasons for delayed diagnosis were lack of equipment to run tests and ignorance. Unsurprisingly, a review study in Africa also highlighted logistical challenges and ignorance among other factors as barriers when screening, diagnosing and managing hyperglycemia in pregnancy [5]. 

Inadequate resources, the second subtheme on resource-centred experiences, was documented as a challenge in GDM care in this study. One HCP recounted instances where OGTT were requested only for pregnant women to bring test results after two or three weeks, citing the cost involved as the reason for the delay. This has the potential to affect treatment, as HCPs are not able to adequately track or monitor blood glucose levels using internationally recommended standards. This further highlights the need to make the OGTT more accessible to pregnant women; this could be through the NHIS, the free maternal health policy or even subsidization of the cost for screening and diagnostic tests. Financial support and subsidization of care have been reported as facilitators of GDM care in Africa [27, 28].

As part of the subtheme on inadequate resources, inadequate staff and logistical challenges were recognized to affect GDM care. The current study noted that due to workload, follow-up is usually difficult. Relatedly, shortage of relevant staff and trained personnel in other African countries have been reported as barriers in GDM care [27, 29]. One HCP in the current study lamented over the absence of test kits to adequately screen and monitor blood sugar levels. Similarly, a study in Ethiopia reported on frequent shortages of logistics and supplies for GDM screening [14]. Also, on lessons learnt from the World Diabetes funded projects in some LMICs (Cameroun, China, Cuban, India, Jamaica, Kenya, and Sudan), poor follow-up systems and absence of test kits were reported as barriers to healthcare [29]. In India, lack of equipment and overcrowding at health facilities have been cited as challenges within the context of GDM screening and management [10]. Hence, there is a need for governments and other relevant stakeholders to regularly provide the resources needed to adequately screen and manage GDM. 

The third subtheme on resource-centred experiences was non-compliance. Unavailability of foods for diet therapy, workload, and poverty were some of the reasons for non-compliance in the current study. Some of the foods prescribed by dieticians become scarce in the dry season, making them costly and inaccessible for some patients, thus threatening compliance to such dietary regimens. In line with the current study findings, environments dominated by unhealthy foods has been recognized as a barrier in GDM care [30]. Moreover, the synergy among the high cost of diagnostic tests for GDM, scarcity of certain foods in the dry season, and inadequate staff or HCPs creates the right atmosphere for non-compliance. This underscores the need for more HCPs to be recruited into healthcare delivery systems, especially dieticians/nutritionists, medical officers, midwives, nurses and laboratory scientists/technicians, to diligently monitor and follow-up on clients and possibly revise management regimen when compliance becomes an issue. For example, dieticians/nutritionists could revise diet plan of clients when prescribed foods become scarce. Similarly, Sinha and colleagues [9] noted that government programs and support groups facilitated the screening and management process after identifying access to healthy foods and insurance issues as a challenge in the screening and management of GDM.

With regard to care-centred experiences, one of the subthemes was raw emotions, HCPs observed that women and their families expressed mixed feelings after learning about GDM status. The HCPs noted that women were emotional about their diagnosis, as they expressed raw emotions, which included worry, shock, confusion, and indifference. Similarly, a couple of studies in Ghana reported on the emotional experiences of patients after being diagnosed with GDM, one of them reported mixed feelings from participants [31], while the other reported worry, confusion and fear [16]. Other studies in the past have also reported feelings of shock when women learnt about their diagnosis [32]. One of the HCPs further indicated that the emotions expressed were mostly dependent on the pregnant woman’s understanding of GDM; those who knew about GDM were worried about themselves and their foetus, and those with little or no knowledge were not bothered or even uncertain about how they felt. A study in China also reported that some women were bothered after being diagnosed with GDM, while others were not [33].

Support from family members and relatives was observed to be integral in modulating the emotional response of patients, particularly those that are hormonal in nature. Support, especially from close relatives, has been reported in previous studies to be essential for managing GDM [34, 35]. Hence, family members could be critical in helping pregnant women follow their dietary regimen despite hormonal changes. However, when family members do not provide the needed support, particularly financial and emotional support, it exacerbates the climate for non-compliance.

The second subtheme on care-centred experiences was non-pharmacological therapy and focused on counselling. Counselling was shown to be a major component of GDM screening and management. HCPs generally admonish pregnant women to engage in self-care behaviours, especially to patronize preconception care and to monitor blood glucose regularly; self-care behaviours have been reported to improve quality of life [36]. The current study revealed that the first line of action when treating or managing GDM is diet and exercise, and oral medications such as metformin and insulin are the last line of action to aid in blood sugar control. This is in line with the Norwegian guidelines for managing GDM [37]. Moholdt and colleagues [38] also recommended a combination of diet and exercise as the first line of action.

The third theme on patient-centred experiences was pharmacotherapy, and comprised medications used to lower blood sugar. Oral medications such as insulin and metformin were used to control blood sugar levels in the current study. HCPs resorted to oral medications when they were not able to control blood sugar with diet and exercise. The most common oral medication used was insulin, which is captured by Garrison’s [13] suggestion that pharmacologic therapy should be initiated with insulin, metformin or glyburide when blood sugar levels are still above target levels after glucose monitoring and lifestyle modifications. Another study showed that physicians initially tried to lower blood sugar levels by using diet and exercise and only used insulin when those methods failed [14]. In addition, the American Diabetes Association’s standards of medical care for diabetes patients recommend that when medical nutrition therapy, exercise, and glucose monitoring are not successful at controlling blood glucose levels, insulin may be given as a last resort [15].

This study is not without limitations; for instance, only five participants were interviewed, which could raise concerns about the generalisability of our findings. This notwithstanding, we believe that the key informants interviewed in this study adequately represent healthcare providers’ experiences because the ANC setups of secondary facilities are similar. In addition, the people interviewed were well versed about GDM screening and management in northern Ghana.

Additionally, HCPs provided their perspectives on patients’ feelings or emotions immediately after GDM diagnosis, which may not necessarily represent how patients felt after the diagnosis. However, we believe the healthcare providers’ perception of how patients felt after diagnosis could affect GDM care and patient compliance. For instance, pregnant women bemoaned the lack of attention and sympathy from HCPs in a study to describe their experiences on being diagnosed and living with GDM [31], while another study reported that HCPs did not have the time to explain diagnosis [30]. Hence, this study provides insights on healthcare providers’ experiences on screening and managing GDM, which could be relevant for advocacy, and social and behavioural change communication on GDM care. It could also be relevant for health planning and policies, because it provides insights into the resources needed to effectively screen and manage GDM.

## Conclusion

This study generated two main themes on healthcare providers’ experiences in the screening and management of GDM, namely: resource-centred experiences, and care-centred experiences. The current findings suggest the need for health system strengthening through the recruitment of more dedicated skilled personnel and government interventions through the revision of existing policies to cover the cost of GDM diagnostic tests. Healthcare providers should also consider the psycho-emotional impact of diagnosis when managing GDM.

## Data Availability

The data supporting the findings of this study can be obtained from the corresponding author upon reasonable request.

## Abbreviations

HCPs: Healthcare Providers
HBA1c: Glycated Haemoglobin
GDM: Gestational Diabetes Mellitus
ANC: Antenatal Care
OGTT: Oral Glucose Tolerance Test
